# Fermented dried *Citrus unshiu* peel extracts exert anti-inflammatory activities in LPS-induced RAW264.7 macrophages and improve skin moisturizing efficacy in immortalized human HaCaT keratinocytes

**DOI:** 10.1080/13880209.2019.1621353

**Published:** 2019-06-12

**Authors:** Chulwon Kim, Jun Ji, Seung Ho Baek, Jong Hyun Lee, In Jin Ha, Soon Sung Lim, Hong Jae Yoon, Yun Je Nam, Kwang Seok Ahn

**Affiliations:** a College of Korean Medicine, Kyung Hee University, Seoul, Republic of Korea;; b Department of Food Science and Nutrition, Hallym University, Chuncheon, Republic of Korea;; c College of Korean Medicine, Woosuk University, Wanju, Korea;; d Korean Medicine Clinical Trial Center (K-CTC), Kyung Hee University Korean Medicine Hospital, Seoul, Republic of Korea;; e Institute of Natural Medicine, Hallym University, Chuncheon, Republic of Korea;; f FA.inc, Republic of Korea

**Keywords:** Inflammation, nitric oxide, cytokine, hyaluronic acid, moisturizing effect

## Abstract

**Context:***Citrus unshiu* Markovich (Rutaceae) peel is known to contain high concentrations of flavonoids and exerts pharmacological effects on antioxidant, anti-inflammation, allergies, diabetes and viral infections.

**Objective:** Very little is known about potential activity of fermented dried *Citrus unshiu* peel extracts (FCU) using *Bacillus subtilis*, as well as its mechanism of action. We investigated the effects of FCU on the anti-inflammatory activities in murine macrophages and moisturizing effects in human keratinocytes.

**Materials and methods:** We isolated the *Bacillus subtilis* from Cheonggukjang and FCU using these *Bacillus subtilis* to prepare samples. The cells were pre-treated with various extracts for 2 h and then induced with LPS for 22 h. We determined the NO assay, TNF-α, IL-6 and PGE_2_ in RAW 264.7 ells. The expression of SPT and Filaggrin by FCU treatment was measured in HaCaT cells.

**Result:** We found that two types of FCU highly suppressed LPS-induced nitric oxide (NO) without exerting cytotoxic effects on RAW 264.7 cells (21.9 and 15.4% reduction). FCU inhibited the expression of LPS-induced iNOS and COX-2 proteins and their mRNAs in a concentration-dependent manner. TNF-α (59 and 30.9% reduction), IL-6 (39.1 and 65.6% reduction), and PGE_2_ secretion (78.6 and 82.5% reduction) were suppressed by FCU in LPS-stimulated macrophages. Furthermore, FCU can induce the production of hyaluronic acid (38 and 38.9% induction) and expression of Filaggrin and SPT in HaCaT keratinocyte cells.

**Discussion and conclusion:** FCU potentially inhibits inflammation, improves skin moisturizing efficacy, and it may be a therapeutic candidate for the treatment of inflammation and dry skin.

## Introduction

Inflammation is a complex process mediated by the activation of various immune cells. Many studies have shown that inflammation is associated with various human diseases, including cancer (Pan and Ho 2008). Macrophages play an important role in various inflammatory responses by upregulating the expression of pro-inflammatory cytokines and enzymes such as tumour necrosis factor-α (TNF-α), interleukin, inducible nitric oxide synthase (iNOS) and cyclooxygenase-2 (COX-2) (Larsen and Henson 1983; Lawrence et al. 2002; Sica et al. 2008). Mechanistically, lipopolysaccharide (LPS), a component of the cell wall of Gram-negative bacteria, interacts with toll-like receptor 4 (TLR4), and triggers the activation of monocytes and macrophages involved in infection response (Aderem and Ulevitch 2000; Saluk-Juszczak and Wachowicz 2005; Takeda and Akira 2005; Pan et al. 2008). Low levels of NO have many biological functions, including neurotransmitters, vascular homoeostasis and wound repair and have antimicrobial activity against bacterial pathogens. NO can be synthesized from L-arginine by a family of NO synthases (NOS). An inducible isoform of NOS (iNOS) is only expressed after exposure to pro-inflammatory conditions. Once expressed, iNOS generates large amounts of NO, which plays an important role in acute and chronic inflammation (Denlinger et al. 1996; Weisz et al. 1996). iNOS is widely expressed in various cells, including vascular smooth muscle cells, hepatocytes and Kupffer cells and is highly expresses in LPS-activated macrophages (Rockey et al. 1998). Cyclooxygenase-2 (COX-2) is another inducible enzyme that catalyses the biosynthesis of prostaglandins (PGEs), particularly PGE_2_, which contributes to pathogenesis of various inflammatory diseases, invasion, angiogenesis, and tumour growth (Claria 2003). COX-2 is also pre-eminently expressed in inflammatory cells stimulated by LPS, pro-inflammatory cytokines and tumour promoters (Meric et al. 2006).

Ceramides are a type of sphingolipid and consist of a sphingoid base and a saturated fatty acid moiety. Ceramides are present as a dominant lipid in the stratum corneum (SC), the most upper layer of the epidermis of the skin and play a crucial role in its water-holding and barrier function (Takeda et al. 2018). Until recently, more than 12 types of ceramide have been designated in human SC (Masukawa et al. 2008). Ceramides in the epidermis are synthesized by several enzymes such as serine palmitoyltransferase (SPT) (Hanada 2003), ceramide synthase (CerS) (Levy and Futerman 2010), glucosylceramide synthase (GCS) (Hamanaka et al. 2002), β-glucocerebrosidese (GBA) (Takagi et al. 1999), sphingomyelin synthase (SMS) (Tafesse et al. 2006), and acid sphingomyelinase (ASM) (Jenkins et al. 2009). SPT and CerS are involved in the *de novo* synthesis of ceramides. SPT catalyses the condensation of serine and palmitoyl-CoA as the first step of *de novo* synthesis. The term `Filaggrin' (derived from `filament-aggregating protein') was first coined in 1981 to describe a class of structural protein that had been isolated from the stratum corneum (Steinert et al. 1981). By aggregating keratin filaments into keratin fibrils within the cytoskeleton of corneocytes, filaggrin is responsible for the mechanical strength and integrity of the stratum corneum (O'Regan et al. 2008). Filaggrin has also been designated as a natural moisturizing factor protein that contributes to the permeable barrier as an aggregated particle comprised of profilaggrin (Kezic et al. 2009).

Plants that have been used worldwide for a long time in traditional medicine have been constantly reviewed as resources for the development of new drugs to control various diseases. Among them, *Citrus unshiu* Markovich (Rutaceae) peel is the dried skin of the *Citrus unshiu* fruit, which is produced primarily in Jeju Island, Korea, and in the southern regions of China and Japan. *Citrus unshiu* peel and dried peels have been used as traditional medicines to treat common colds, bronchial discomfort, and indigestion and have been reported to possess pharmacological effects on inflammation, allergies, diabetes, and viral infections (Suzuki et al. 2005; Oh et al. 2012; Park et al. 2013, 2017; Min et al. 2014). Here, we have investigated an herbal agent that can help skin moisturization and anti-inflammation by using fermented dried *Citrus unshiu* peel extracts. In order to use natural products as raw materials for cosmetics, researchers have primarily investigated potential to improve skin moisturization and block inflammation (Kim et al. 2005; Eom et al. 2006; Spilioti et al. 2017; Shen et al. 2018). However, the effects of fermented *Citrus unshiu* peel extracts on RAW 264.7 mouse macrophage-mediated inflammation and moisturizing effect in HaCaT keratinocytes remain unknown.

In our study, we attempted to elucidate the anti-inflammatory potential of fermented *Citrus unshiu* peel extracts in LPS-stimulated RAW 264.7 macrophages. Here, we evaluated the inhibitory effect of fermented *Citrus unshiu* peel extracts on inflammatory biomarkers such as NO and PGE_2_ production, expression of iNOS, COX-2, and pro-inflammatory cytokines in LPS-stimulated RAW 264.7 cells. Furthermore, fermented *Citrus unshiu* peel extracts can stimulate the production of hyaluronic acid and expression of filaggrin and SPT in HaCaT keratinocyte cells, which is an indicative of moisturizing effect.

## Materials and methods

### Reagents

LPS (*Escherichia coli* 055:B5), 3-(4,5-dimethylthiazol-2-yl)-2,5-diphenyltetrazolium bromide (MTT), Tris base, glycine, NaCl, sodium dodecylsulphate (SDS), Griess reagent and bovine serum albumin (BSA) were purchased from Sigma-Aldrich (St. Louis, MO, USA). RPMI 1640, foetal bovine serum (FBS) and antibiotic-antimycotic mixture were obtained from Thermo Fisher Scientific Inc. (Waltham, MA, USA). Trypan blue vital stain (0.4%) was obtained from Life Technologies (Grand Island, NY, USA). PGE_2_, TNF-α, IL-6 ELISA kits were obtained from R&D Systems (Minneapolis, MN, USA). Anti-COX-2 and anti-iNOS antibodies were obtained from BD Biosciences (San Diego, CA, USA). Anti-filaggrin, anti-serine palmitoyltransferase (SPT), anti-β-actin, and horseradish peroxidase (HRP)-conjugated secondary antibodies were obtained from Santa Cruz Biotechnology (Santa Cruz, CA, USA).

### Isolation of *Bacillus subtilis* from Korean fermented soybean (cheonggukjang)

Samples of Cheonggukjang (1 mL), serially diluted, were plated on tryptic soy broth (TSB) agar (Becton-Dickinson, Franklin Lakes, NJ, USA) and placed at 30 °C for 24 h under anaerobiosis to isolate presumptive mesophilic *Bacillus subtilis*. At least 10 colonies, possibly with different morphology, were isolated on the TSB plates. Two *Bacillus subtilis* were identified by TLC on activated silica gel plates using *n*-butanol: acetic acid: water (5:2:2, v/v/v). Isolated *Bacillus subtilis* strains were named ‘1-4’ and ‘2-1’, respectively.

### Preparation of FCU using *Bacillus subtilis*

The isolated *Bacillus subtilis* strains (‘1-4’ and ‘2-1’) were cultured in tryptic soy broth (TSB) at 37 °C. For the production of fermented dried *Citrus unshiu* peel, we first added water to the dried *Citrus unshiu* peel at a ratio of 1:20 (w/v) and heated for 2 h at 95 °C. After autoclaving at 121 °C for 15 min, a dried *Citrus unshiu* peel extracts were obtained and the *Bacillus subtilis* (‘1-4’ and ‘2-1’) culture broth was added to the extracts at a concentration of 10% (v/v), which was well mixed and then incubated for 96 h at 37 °C. The fermented extracts were filtered using an aspirator, water and ethanol were added, and filtration was performed again. The supernatant was then dried and stored at 4 °C. The intact extracts (control) had no *Bacillus subtilis*. The sample table for each fermentation condition is shown in Table 1.

**Table 1.** Sample table for each fermentation condition.

Name of sample	*Bacillus subtilis*	Filtrate solution
WE	None	H_2_O
WE(1-4)	1-4 strain	H_2_O
WE(2-1)	2-1 strain	H_2_O
AL	None	EtOH
AL(1-4)	1-4 strain	EtOH
AL(2-1)	2-1 strain	EtOH

### Cell lines

The RAW 264.7 macrophage and HaCaT cell lines were obtained from Korean Cell Line Bank (KCLB, Seoul National University College of Medicine, 28 Yongon-dong, Chongno-gu, Seoul 110-744, Korea). These cells were maintained at subconfluence in a 95% air, 5% CO_2_ humidified atmosphere at 37 °C. RAW 264.7 cells were cultured in RPMI 1640 medium containing 10% FBS. HaCaT cells were cultured in Dulbecco’s Modified Eagle’s medium (DMEM) containing 10% FBS. All media were also supplemented with 100 U/mL of penicillin and 100 μg/mL of streptomycin.

### MTT assay

Cell viability was measured by an MTT assay to detect NADH-dependent dehydrogenase activity. Thirty microliters of MTT solution (2 mg/mL) in 1× phosphate-buffered saline (PBS) was directly added to the cells, which were then incubated for 3 h to allow MTT to metabolize to formazan. Absorbance was measured with an automated spectrophotometric plate reader at a wavelength of 570 nm. Cell viability was normalized as relative percentages in comparison with untreated controls.

### Nitrite assay

The RAW 264.7 macrophage cells were plated at a density of 2 × 10^5^ cells per well in a 24-well plate. The cells were pre-treated with the indicated concentrations of various extracts for 2 h, and then induced with a 1 μg/mL concentration of LPS for an additional 22 h. Nitrite accumulation in the culture was measured colorimetrically by the Griess reaction using a Griess reagent (Sigma-Aldrich, St. Louis, MO, USA). For the assay, equal volumes of cultured medium and Griess reagent were mixed, and the absorption coefficient was calibrated using a sodium nitrite solution standard (Sigma-Aldrich, St. Louis, MO, USA). The absorbance of each sample after the Griess reaction was determined by an ELISA plate reader at 540 nm.

### Measurement of PGE_2_ release by the RAW 264.7 macrophage cells

The RAW 264.7 macrophage cells were plated at a density of 2 × 10^5^ cells per well in a 24-well plate. The cells were pre-treated with the indicated concentrations of WE(2-1) and AL(2-1) for 2 h, and then induced with 1 μg/mL of LPS for an additional 22 h. The level of PGE_2_ production from endogenous arachidonic acid metabolism was measured in cell culture supernatants of the RAW 264.7 cells by enzyme-linked immunosorbent assay (ELISA) kit (R&D Systems, Minneapolis, MN, USA).

### Measurement of pro-inflammatory cytokines (TNF-α and IL-6) production

The inhibitory effect of WE(2-1) and AL(2-1) on the production of proinflammatory cytokines (TNF-α and IL-6) from LPS-treated RAW 264.7 cells was determined. The supernatants were subsequently employed for the proinflammatory cytokine assays using a mouse enzyme-linked immunosorbent assay (ELISA) kit (R&D Systems, Minneapolis, MN, USA).

## Real-time PCR

Real-time quantitative PCR was performed using the Universal SYBR Green Master Mix (Applied Bio systems, USA). Amplification of the cDNA was performed as follow: 95 °C for 15 min followed by 40 cycles at 95 °C for 30 s, at 59 °C for 30 s, and at 72 °C for 30 s. The real-time PCR analysis was performed on an Applied Bio-systems StepOne system (Applied Bio-systems, USA). In this study, quantification based on the relative expression of a target gene versus GAPDH gene (2^−ΔΔCt^) was utilized to determine the level of mRNA expression.

### Reverse transcription polymerase chain reaction (RT-PCR)

Cells were washed and suspended in Trizol reagent. Total RNA was extracted according to the manufacturer’s instructions (Invitrogen, Life Technologies, Carlsbad, CA, USA). One microgram of total RNA was converted to cDNA by superscript reverse transcriptase and then amplified by a Taq polymerase using reverse transcription polymerase chain reaction (RT-PCR) (TAKARA, Tokyo, Japan). The relative expression of iNOS, COX-2, filaggrin, and SPT were analyzed using PCR with glyceraldehyde-3-phosphate dehydrogenase (GAPDH) as an internal control. The following pairs of forward and reverse primer sets were used: iNOS, 5′-TCTTTGACGCTCG-GAACTGTAGCA-3′ and 5′-CGTGAAGCCATGACCTTTCGCATT-3′. COX-2, 5′-TTGCTGTACAAGCAGT-GGCAAAGG-3′ and 5′-AGGACAAACACCGGAGGGAATCTT-3′. Filaggrin, 5′-CAAATCCTGAAGAATCCAGATGAC-3′ and 5′-TGCTTGAGCCAACTTGAATACC-3′, serine-palmitoyltransferase, 5′-TTTCCGGTTTAAAAGTGGTG-3′ and 5′-CTGATGCTTGGAGGAGGAAG-3′. The cDNA reaction was performed at 45 °C for 60 min and 95 °C for 5 min. PCR products were run on 1% agarose gel and then stained with Loading Star (Dynebio, Seongnam, Korea). Stained bands were visualized under UV light and photographed.

### Western blotting

After the cells were treated with the indicated concentrations of WE(2-1) and AL(2-1), the cells were lysed and the total protein concentrations were determined by Bradford reagent (Bio-Rad, Hercules, CA, USA). Equal amounts of lysates resolved on sodium dodecyl-polyacrylamide gel electrophoresis (SDS-PAGE) were transferred to a nitrocellulose membrane, and the membrane was blocked with 1× TBS containing 0.1% Tween 20 and 5% skim milk or 2% BSA for 1 h at room temperature. After the blocking, the membranes were incubated overnight at 4 °C with the respective primary antibodies. The membranes were washed twice and incubated with diluted horseradish peroxidase (HRP)-conjugated secondary antibodies (1:10000) for 1 h at room temperature. After three washes, the membranes were detected using an enhanced chemiluminescence (ECL) kit (Millipore, Bedford, MA, USA).

### Monitoring of cell growth with the RTCA DP instrument

Cell growth behavior was continuously monitored for 120 h using the xCELLigence RTCA DP Instrument (Roche Diagnostics GmbH, Germany). Background impedance was measured in 100 μL cell culture medium per well. The final volume was adjusted to 200 μL cell culture medium, including 5 × 10^3^ cells/well. After plating, impedance was recorded in 15 min intervals. All experiments were performed in triplicates. Cell Index (CI) values were normalized to the time point of indicated concentration of WE(2-1) and AL(2-1) administration (referred to as normalized CI).

### Hyaluronic acid measurement

The cellular hyaluronic acid levels were measured by Hyaluronan Quantikine ELISA Kit (R&D Systems) according to the manufacturer’s protocol.

### Analysis of flavonoids in WE(2-1) and AL(2-1)

The chemical profiles of the WE(2-1), AL(2-1) and the chemical changes of constituents were monitored by the liquid chromatography-mass spectrometry system consisted of a Thermo Scientific Vanquish UHPLC system (Thermo Fisher Scientific, Sunnyvale, CA, USA) with a Shim-pack GIS-ODS (3 µm, 3.0 × 100 mm, Shimadzu) and a Triple TOF 5600+ mass spectrometer system (Triple TOF MS; QTOF, SCIEX, Foster City, CA, USA). The QTOF MS, equipped with a Duospray™ ion source, and was used to complete the high-resolution experiment. The LC gradient used a mobile phase a containing 0.1% formic acid in water and a mobile phase B of acetonitrile with 0.1% formic acid. The flow rate was kept constant at 0.8 mL/min and the injection volume was 2 μL. The gradient elution system was as follows: 5–100% B for 12 min. Authentic standards; narirutin, narigenin, rutin, hesperidin, hesperetin, neoponcirin, tangeretin, and nobiletin were purchased from Chemface (Wuhan, China). HPLC-grade acetonitrile (ACN) and methanol (MeOH) were purchased from Honeywell Burdick & Jackson (Morristown, NJ, USA). Analytical-grade formic acid (99% purity) was obtained from Sigma-Aldrich (St. Louis, MO, USA). The chemical profiles of six extracts were analyzed and compared using LC-QTOF. Eight major bioactive components were identified with chromatographic information (retention time, mass; Da, and fragmentation ions) of authentic standards. Data acquisition and processing were carried out using Analyst TF 1.7, PeakVeiw 2.2 and MasterView software (SCIEX, Foster City, CA, USA).

LC: Liquid chromatography, QTOF: Quadurpole Time-of-Flight mass spectrometry.

### Statistical analysis

All numeric values are represented as the mean ± SE. Statistical significance of the data compared with the untreated control was determined using the Mann–Whitney U-test. Significance was set at *p* < 0.05.

## Results

### Cytotoxic effect of the six FCU in RAW 264.7 macrophages

**Figure 1.**
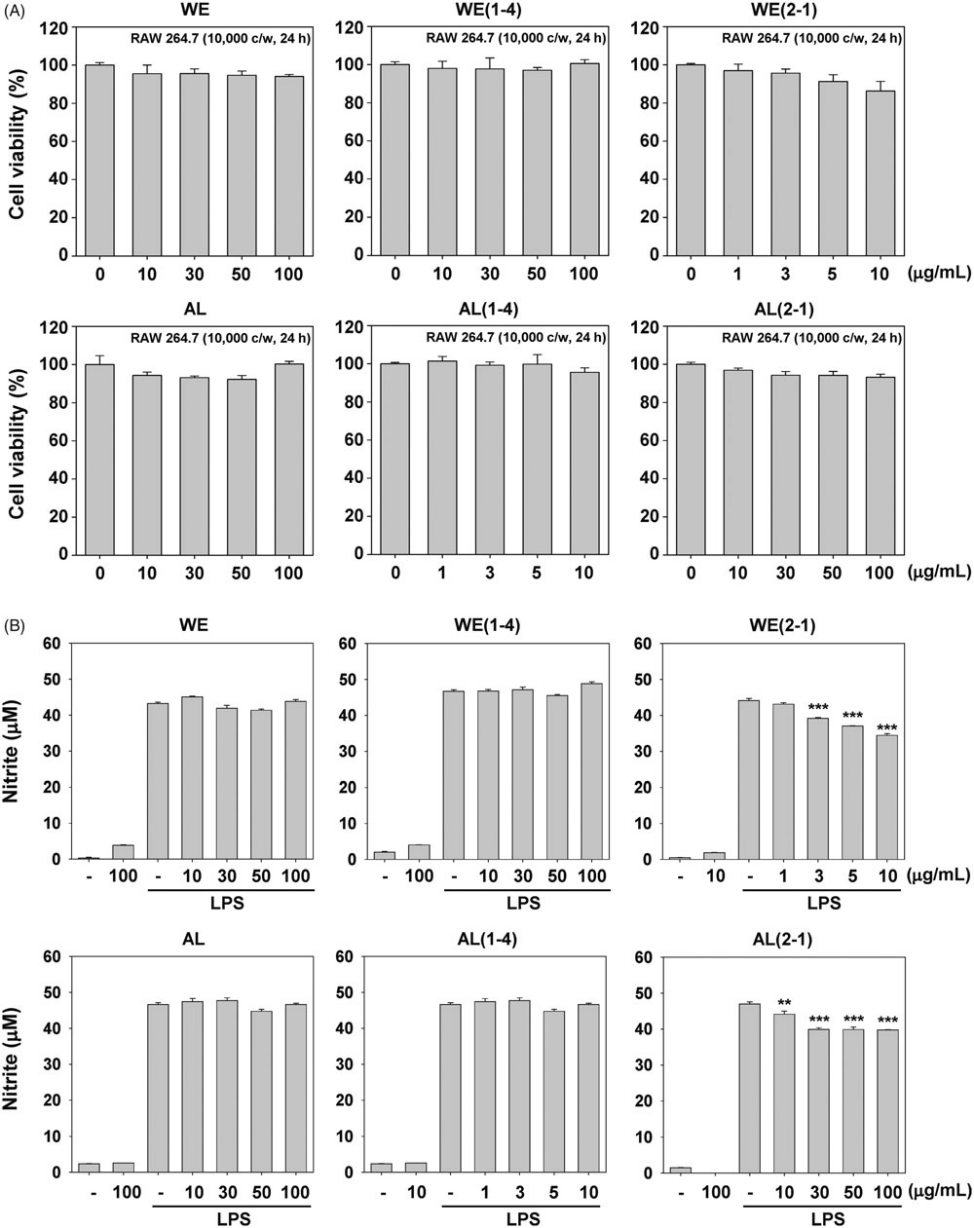
Inhibition of the WE(2-1) and AL(2-1) on LPS-induced nitric oxide (NO) in RAW 264.7 macrophages. (A) RAW 264.7 cells (1 × 10^4^ cells/well) were treated with the indicated concentrations of various extracts for 24 h and cell viability was determined by MTT assay. Results of independent experiments were averaged and are shown as percentage cell viability compared with the viability of untreated control cells. (B) The nitrite production was measured by the Griess reaction assay method as described in the methods section. Cells were pre-treated with indicated concentrations of various extracts for 2 h and stimulated with LPS (1 µg/mL) for 22 h. The values obtained were compared with those of standard concentrations of sodium nitrite dissolved in RPMI 1640 medium, and the concentrations of nitrite in a conditioned media of sample treated cells were calculated. Data were obtained from three independent experiments and were expressed as means ± SD. ***p < 0.001 indicates significant differences from the unstimulated control group.

We investigated for the first time the anti-inflammatory effects of FCU in RAW 246.7 macrophages. Here, we examined the cytotoxic effects of six FCU and cell viability was assessed using the MTT assay. We found that WE, WE(1-4), AL, and AL(1-4) had no effect on the cytotoxicity except for WE(2-1) and AL(2-1) at a concentration of 10 or 100 μg/mL (Figure 1(A)).

### Inhibition of LPS-induced NO production by six fermented extracts in RAW 264.7 macrophages

In order to investigate the anti-inflammatory effects of six fermented extracts, we first investigated its effects on nitrite production in LPS-stimulated RAW 264.7 macrophages. The effects of six fermented extracts on LPS-induced NO production in RAW 264.7 macrophages were investigated by measuring the accumulated nitrite in the culture medium as estimated by the Griess reaction. Cells were pre-treated with the indicated concentrations of six fermented extracts for 2 h and then induced with LPS (1 μg/mL) for 22 h. LPS-treated cells significantly increased the concentration of NO. As shown in Figure 1(B), in cells which were pre-treated with various concentrations of six fermented extracts and also together with 1 μg/mL of LPS for 22 h, significant concentration-dependent inhibition of nitrite production was found in WE(2-1) and AL(2-1) treated cells.

### Inhibition of LPS-induced PGE_2_ secretion by WE(2-1) and AL(2-1) in RAW 264.7 cells

**Figure 2.**
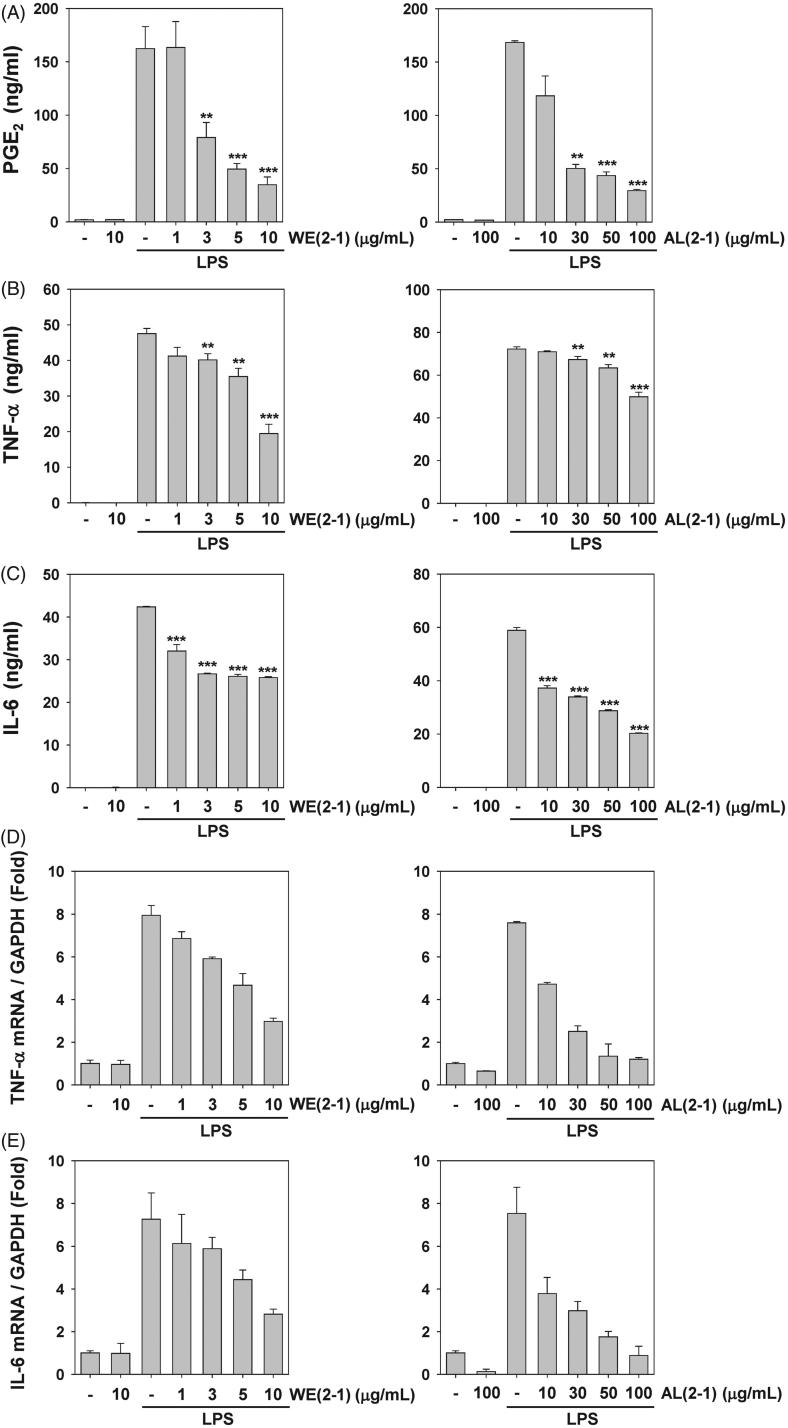
Inhibition of LPS-induced several inflammatory biomarkers by WE(2-1) and AL(2-1) in RAW 264.7 macrophages. (A) Cells were pre-treated with different concentrations of WE(2-1) and AL(2-1) for 2 h and stimulated with LPS (1 µg/mL final concentration) for 22 h. The amount of PGE_2_ release was determined by the mouse PGE_2_ ELISA kit. Data were obtained from three independent experiments and were expressed as means ± SD. **p < 0.01 and ***p < 0.001 indicate significant differences from the LPS-treated group. (B) Cells were pre-treated with different concentrations of WE(2-1) and AL(2-1) for 2 h and stimulated with LPS (1 µg/mL final concentration) for 22 h. The amount of TNF-α release was determined by a TNF-α antibody-coated enzyme-linked immunosorbent assay (ELISA) kit following the manufacturer’s instructions. Data were obtained from three independent experiments and were expressed as means ± SD. **p < 0.01 and ***p < 0.001 indicate significant differences from the LPS-treated group. (C) Cells were pre-treated with different concentrations of WE(2-1) and AL(2-1) for 2 h and stimulated with LPS (1 µg/mL final concentration) for 22 h. The amount of IL-6 release was determined by an IL-6 antibody coated ELISA kit following the manufacturer’s instructions. Data were obtained from three independent experiments and were expressed as means ± SD. **p < 0.01 and ***p < 0.001 indicate significant differences from the LPS-treated group. (D and E) Cells were pre-treated with different concentrations of WE(2-1) and AL(2-1) for 2 h and stimulated with LPS (1 µg/mL final concentration) for 22 h. The total RNA was isolated, and examined via Real-time PCR for the TNF-α and IL-6 genes. Glyceraldehyde-3-phosphate dehydrogenase (*GAPDH*) was employed as an internal control to demonstrate equal RNA loading.

To examine the potential anti-inflammatory properties of WE(2-1) and AL(2-1) on LPS-induced prostaglandin E_2_ (PGE_2_) production in RAW 264.7 cells, cells were pre-treated with or without WE(2-1) (1, 3, 5, 10 μg/mL) and AL(2-1) (10, 30, 50, 100 μg/mL) for 2 h and then stimulated with LPS (1 μg/mL) for 24 h. PGE_2_ concentrations were measured in the culture supernatants by the ELISA assays. We found that WE(2-1) and AL(2-1) substantially suppressed LPS-induced PGE_2_ production in a concentration-dependent manner (Figure 2(A)).

### Inhibition of LPS-induced pro-inflammatory cytokines by WE(2-1) and AL(2-1) in RAW 264.7 cells

Pro-inflammatory cytokines such as TNF-α, and IL-6 play important roles in immune responses to a variety of inflammatory stimuli. Therefore, the inhibitory effect of WE(2-1) and AL(2-1) on TNF-α, and IL-6 in LPS-stimulated RAW 264.7 cells were evaluated using ELISA kits. The treatment of RAW 264.7 cells with LPS alone resulted in significant increases in these cytokine production, however, WE(2-1) and AL(2-1) pre-treated cell repressed LPS-induced TNF-α, and IL-6 production in a concentration-dependent manner (Figure 2(B and C)). When RNA was isolated and quantitative real-time PCR was performed to examine the effects of WE(2-1) and AL(2-1) on gene expression, treatment of RAW264.7 cells with LPS alone showed a significantly increases in TNF-α and IL-6 mRNA expression. In the co-incubation of WE(2-1) with LPS, TNF-α expression level was found to decrease from 7.9- to 7.2-, 5.1-, 4.2-, 2.1-fold in a concentration-dependent manner as compared with the LPS alone treatment group. In the co-incubation of AL(2-1) with LPS, TNF-α expression level was found to decrease from 7.5- to 4.7-, 2.5-, 1.3-, 1.2-fold in a concentration-dependent manner (Figure 2(D)). In the co-incubation of WE(2-1) with LPS, IL-6 expression level was found to decrease from 7.2- to 6.1-, 5.8-, 4.4-, 2.8-fold in a concentration-dependent manner as compared with the LPS alone treatment group. In the co-incubation of AL(2-1) with LPS, IL-6 expression level was found to decrease from 7.5- to 3.7-, 2.9-, 1.7-, 0.8-fold in a concentration-dependent manner (Figure 2(E)).

### Inhibition of LPS-induced iNOS and COX-2 protein and its mRNA expression by WE(2-1) and AL(2-1)

**Figure 3.**
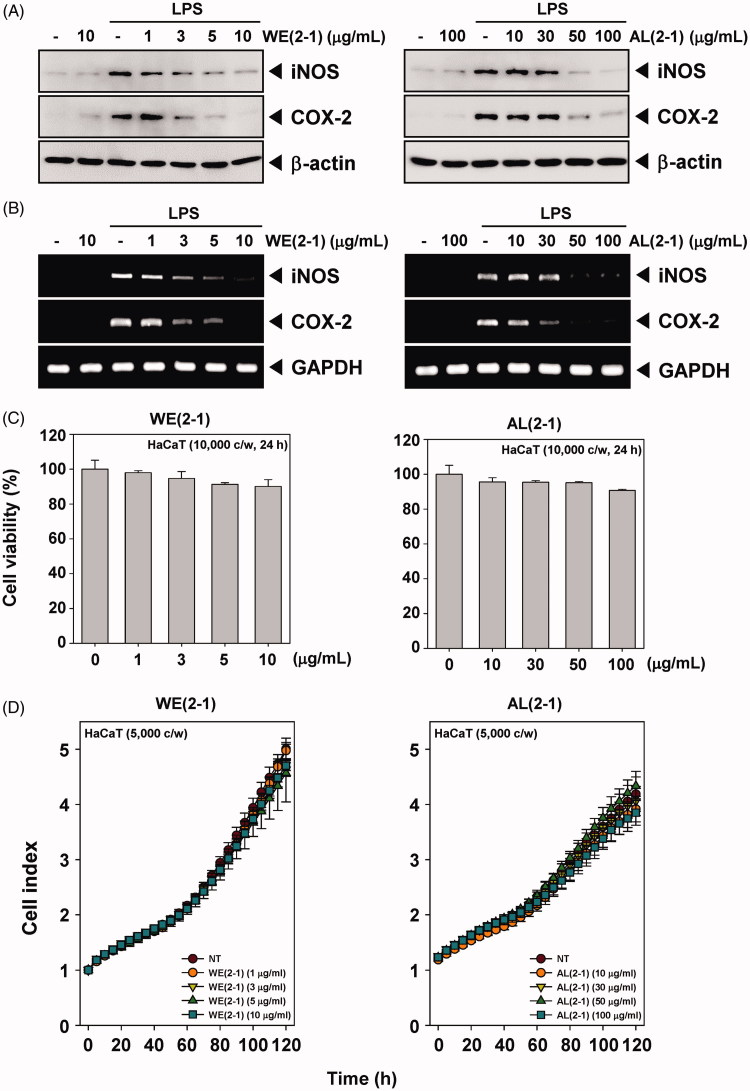
Inhibition of the WE(2-1) and AL(2-1) on LPS-induced iNOS, and COX-2 gene products in RAW 264.7 macrophages. (A) RAW 264.7 cells were pre-treated with the indicated concentrations of WE(2-1) and AL(2-1) for 2 h before being incubated with LPS (1 μg/mL) for 22 h. Total RNA was isolated, and iNOS and COX-2 mRNA expressions were examined by RT-PCR analysis. PCR of glyceraldehydes-3-phosphatedehydrogenase, GAPDH, was performed to control for a similar initial cDNA content of the sample. The results shown are representative of the three independent experiments. (B) RAW 264.7 cells were pre-treated with different concentrations of WE(2-1) and AL(2-1) for 2 h and stimulated with LPS (1 µg/mL) for 22 h. Equal amounts of total proteins (10 μg/lane) were subjected to 10% SDS-PAGE, and the expressions of iNOS and COX-2 proteins were detected by Western blotting using specific anti-iNOS and anti-COX-2 antibodies. β-actin was used as a loading control. The blots shown are representative of three independent experiments that had similar results. (C) HaCaT cells (1 × 10^4^ cells/well) were treated with the indicated concentrations of WE(2-1) and AL(2-1) for 24 h and cell viability was determined by MTT assay. Results of independent experiments were averaged and are shown as percentage cell viability compared with the viability of untreated control cells. (D) Cell proliferation assay was performed using the Roche xCELLigence Real-Time Cell Analyzer (RTCA) DP instrument (Roche Diagnostics GmbH, Germany) as described in ‘Material and methods’. After HaCaT cells (5 × 10^3^ cells/well) were seeded onto 16-well E-plates and continuously monitored using impedance technology.

The effects of WE(2-1) and AL(2-1) on iNOS and COX-2 protein expression in RAW 264.7 macrophages were examined by Western blot analysis. As shown in Figure 3(A), the cells expressed extremely low detectable levels of iNOS and COX-2 proteins under an unstimulated condition; however, iNOS and COX-2 expressions were highly increased in response to LPS (1 μg/mL) after 22 h. Pretreatment of cells with WE(2-1) (1, 3, 5, 10 μg/mL) and AL(2-1) (10, 30, 50, 100 μg/mL) for 2 h dramatically suppressed LPS-induced iNOS and COX-2 expression in a concentration-dependent manner (Figure 3(A)). The individual treatment of WE(2-1) (10 μg/mL) and AL(2-1) (100 μg/mL) alone did not affect the basal iNOS and COX-2 expressions.

Also, we have attempted to determine whether the suppression in the expression of iNOS and COX-2 proteins paralleled their inhibition at mRNA levels. After a 2 h pre-treatment of WE(2-1) and AL(2-1), RAW 264.7 macrophages were stimulated with LPS for 22 h. Then, they were harvested and assayed for iNOS and COX-2 mRNA expressions by RT-PCR. The results demonstrate the upregulation of its mRNA levels upon stimulation with LPS for 22 h. In unstimulated macrophages, there was no detectable mRNA. Pre-incubation of cells with WE(2-1) and AL(2-1) plus LPS caused a suppression of iNOS and COX-2 mRNA induction after 22 h of incubation. RT-PCR analysis showed that WE(2-1) and AL(2-1) suppressed the LPS-induced iNOS and COX-2 mRNA levels in a concentration-dependent manner (Figure 3(B)).

### Cytotoxic effect of the WE(2-1) and AL(2-1) in HaCaT keratinocyte cells

We next examined the cytotoxic effects of WE(2-1) and AL(2-1) and cell viability was assessed using the MTT assay. We found that WE(2-1) and AL(2-1) had little effect on the cytotoxicity in HaCaT cells (Figure 3(C)).

### WE(2-1) and AL(2-1) had little effect on cell proliferation in HaCaT cells

To specifically examine the cell proliferation activity of WE(2-1) and AL(2-1) on HaCaT cells, the cells were treated with indicated concentrations of WE(2-1) and AL(2-1), and then cell viability was analyzed every 15 min time intervals using the xCELLigence RTCA DP Instrument (Roche Diagnostics GmbH, Germany). As shown in Figure 3(D), WE(2-1) and AL(2-1) had little effect on cell proliferation in HaCaT cells in a time-dependent manner.

### WE(2-1) and AL(2-1) stimulate the production of hyaluronic acid in HaCaT cells

**Figure 4.**
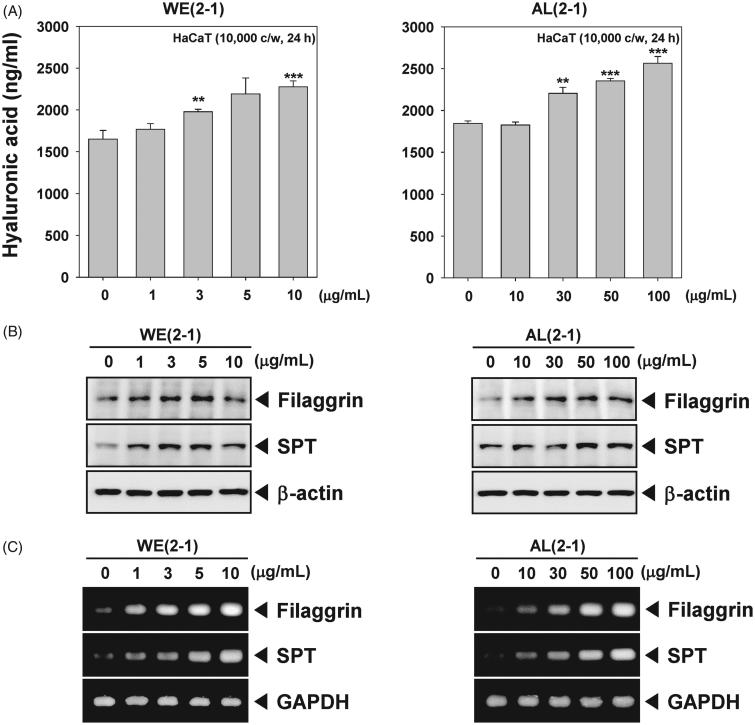
WE(2-1) and AL(2-1) stimulates filaggrin and serine palmitoyltransferase (SPT). (A) HaCaT cells (1 × 10^6^ cells/well) were exposed to indicated concentrations of WE(2-1) and AL(2-1) for 24 h, and then supernatant collected for investigation using a Hyaluronic acid assay kit following the manufacturer’s instructions. (B) HaCaT cells (1 × 10^6^ cells/well) were treated with the indicated concentrations of WE(2-1) and AL(2-1) for 24 h. Total RNA was isolated, and filaggrin and SPT mRNA expressions were examined by RT-PCR analysis. PCR of glyceraldehydes-3-phosphatedehydrogenase, GAPDH, was performed to control for a similar initial cDNA content of the sample. The results shown are representative of the three independent experiments. (C) HaCaT cells (1 × 10^6^ cells/well) were treated with indicated concentrations of WE(2-1) and AL(2-1) for 24 h and then analyzed by Western blot analysis using antibodies against filaggrin, and SPT. β-actin was used as a loading control. The blots shown are representative of three independent experiments that had similar results.

We next set out to determine the effect of WE(2-1) and AL(2-1) on hyaluronic acid production in HaCaT cells. Hyaluronic acid is known to act as a sponge in the skin to attract and hold water (Draelos 2012). After treatment for 24 h, WE(2-1) and AL(2-1)-induced an increased production of hyaluronic acid, which is an indicative of moisturizing effect (Figure 4(A)).

### WE(2-1) and AL(2-1) induces the expression of filaggrin and SPT in HaCaT cells

Filaggrin and SPT acting as a key factor for skin hydration, we examined whether WE(2-1) and AL(2-1) regulates the expression of these proteins. As shown in Figure 4(B), WE(2-1) and AL(2-1) led to increased expression of filaggrin and SPT in HaCaT cells at the protein level. WE(2-1) and AL(2-1) also enhanced mRNA level of filaggrin and SPT in a concentration-dependent manner in HaCaT cells (Figure 4(C)).

### Identification of flavonoids in WE(2-1) and AL(2-1)

**Figure 5.**
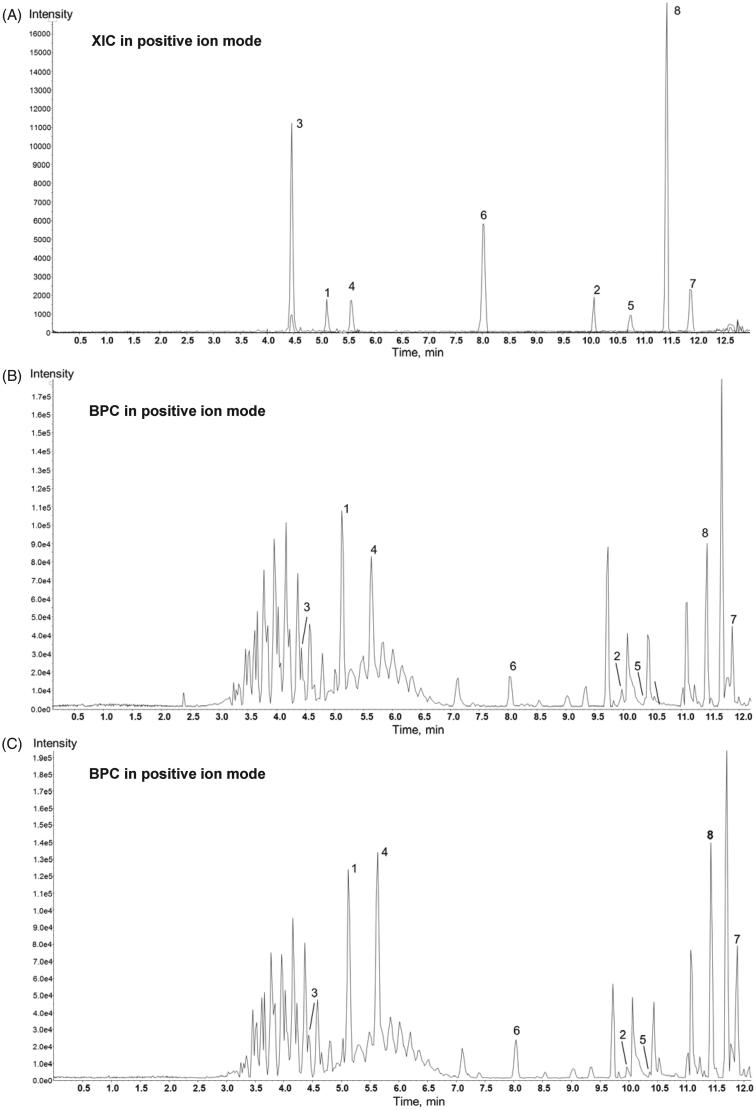
Chemical profiles of the authentic standards, WE(2-1), and AL(2-1). (A) LC-QTOF extracted ion chromatography (XIC) of 8 authentic standards; **1** narirutin, **2** narigenin, **3** rutin, **4** hesperidin, **5** hesperetin, **6** neoponcirin, **7** tangeretin, **8** nobiletin and (B) representative base peak ion chromatograms (BPC) of WE(2-1) and **(C)** AL(2-1).

Among flavonoids, dominant flavanone glycosides in all six samples are three flavanone glycosides (hesperidin, narirutin, and neoponcirin) identified by comparing retention times of analytes in authentic standards and samples (Figure 5(A)), as well as according to the characteristic exact mass and tandem mass (ms/ms) spectra. Hesperidin and narirutin are well known as analytical markers of *Citrus unshiu* peel. Rutin flavonol glycoside was identified in all samples. Polymethoxyflavones (nobiletin and tangeretin) and flavanones (hesperetin and narigenin) were authentically identified. As shown in Figure 5(B) and (C), the peak between nobiletin and tangeretin at 11.70 min is putatively identified as heptamethoxyflavone. The list of major compounds detected by LC-QTOF is shown in Table 2.

**Table 2.** List of major compounds detected by LC-QTOF.

Compound	Formula	Retention Time (min)	[M + H]^+^ Mass (Da)	[M + H]^+^ Found At Mass (Da)
Narirutin	C_27_H_32_O_14_	5.06	581.1865	581.1851
Narigenin	C_15_H_12_O_5_	9.99	273.0758	273.0751
Rutin	C_27_H_30_O_16_	4.41	611.1607	611.1590
Hesperidin	C_28_H_34_O_15_	5.57	611.1971	611.1955
Hesperetin	C_16_H_14_O_6_	10.29	303.0863	303.0856
Neoponcirin	C_28_H_34_O_14_	7.97	595.2021	595.2020
Tangeretin	C_20_H_20_O_7_	11.86	373.1282	373.1280
Nobiletin	C_21_H_22_O_8_	11.42	403.1387	403.1383

## Discussion

The aim of this study was to investigate the effects of FCU on the suppression of LPS-induced iNOS, COX-2 expression, TNF-α, IL-6, and PGE_2_ production that regulate inflammation in RAW 264.7 murine macrophages. Moreover, fermented *Citrus unshiu* peel extracts can cause stimulate the production of hyaluronic acid and expression of filaggrin and SPT in HaCaT keratinocyte cells, which is an indicative of moisturizing effect.

Inflammation is the body's first response of the immune system to infection or irritation. During the inflammatory process, measurable quantities of the inflammatory mediators NO and PGE_2_ are regulated by the inducible isoforms of iNOS and COX-2 (Posadas et al. 2000). Overproduction of NO by iNOS occurred in various cell types after stimulation with cytokine and endotoxin, and are also involved in different inflammatory diseases and tumorigenesis (Nathan and Xie 1994; Ohshima and Bartsch 1994). High levels of PGE_2_ synthesized by COX-2 also occurred in various cancer tissues and implicated in angiogenesis, proliferation, and tumour growth (Claria 2003; Meric et al. 2006). Thus, there is a causal relationship between inflammation and cancer, iNOS and COX-2 are considered potential candidates for anti-inflammatory drugs (Pan et al. 2009). We demonstrated that FCU inhibited LPS-induced NO production via the suppression of iNOS expression. In addition, in an LPS-stimulated macrophage cells, FCU also suppressed PGE_2_ production through the suppression of COX-2 expression. Several studies have demonstrated that the expression of iNOS is induced by pro-inflammatory cytokines, such as IL-1β and TNF-α and TNF-α-induced IL-6 secretion is a prerequisite for increased NO production (Marcus et al. 2003; Schrader et al. 2007). They are also considered to be important initiators of the inflammatory response and mediators of the development of a variety of inflammatory diseases (Glauser 1996; Mannel and Echtenacher 2000). We found that FCU results in a concentration-dependent decrease in the LPS-induced secretion of TNF-α, and IL-6. This indicates that the inhibitory effect of FCU on NO production stimulated by LPS is associated with the inhibition of iNOS and COX-2 through the reduction of pro-inflammatory cytokine production.

The skin barrier function is essential and important to maintain the homoeostasis of the skin because of its role in suppressing the penetration of chemicals and the invasion of microorganisms from the outside environment and also to its role in reducing water evaporation from inside the body. Filaggrin is a key structural protein that promotes the formation of the stratum corneum (SC), the outermost layer of the skin, and the terminal differentiation of the epidermis (Armengot-Carbo et al. 2015). The stratum corneum is essential to minimize water loss through the epidermis and prevent entry of pathogens, allergens and toxic chemicals (O'Regan et al. 2009; Osawa et al. 2011). Filaggrin is initially synthesized as a giant inactive precursor protein, profilaggrin, a > 400-kDa in human, which is a complex, highly phosphorylated, and insoluble polypeptide (Sandilands et al. 2009; Brown and McLean 2012). Filaggrin is degraded into amino acids, including histidine and glutamine, which are subsequently modified into trans-urocanic acid and pyrrolidone carboxylic acid, respectively. These amino acids and derivatives contribute to the formation of natural moisturizing factors. They bind water in the stratum corneum and limit moisture loss in the skin, as well as acidify the skin mantle and protect the epidermis (McAleer and Irvine 2013). The first step in *de novo* biosynthesis of ceramides is catalyzed by the serine palmitoyl transferase (SPT), which is a condensation reaction of L-serine with palmitoyl-CoA. SPT has been suggested as a key enzyme controlling the level of sphingolipid involved in ceramide generation (Hanada 2003). We found that FCU results in a concentration-dependent increases mRNA level of filaggrin and SPT. This can cause stimulate the production of hyaluronic acid and expression of filaggrin and SPT proteins in HaCaT keratinocyte cells. Hyaluronan, an extracellular matrix component, is important for the water content of the skin. So, FCU increases the components which manage the barrier function and water content of the skin.

## Conclusions

Overall, our data clearly indicates that FCU exhibits an anti-inflammatory activity that is dependent on its ability to regulate the production of NO, PGE_2_, and pro-inflammatory cytokines in LPS-induced RAW 264.7 cells. Also, we found the FCU increases filaggrin and SPT on HaCaT cells that play a key role in skin hydration and integrity and are involved in skin appearance, metabolism, mechanical properties, and barrier function.
